# Are German family practitioners and psychiatrists sufficiently trained to diagnose and treat patients with alcohol problems?

**DOI:** 10.1186/s12875-019-1006-8

**Published:** 2019-08-15

**Authors:** T. Hoffmann, K. Voigt, J. Kugler, L. Peschel, A. Bergmann, H. Riemenschneider

**Affiliations:** 1 Department of Health Sciences / Public Health, Technische Universität Dresden, Fetscherstr 74, 01307 Dresden, Germany; 2 Department of General Practice, Medical Clinic 3, Faculty of Medicine Carl Gustav Carus, Technische Universität Dresden, Fetscherstr 74, 01307 Dresden, Germany

**Keywords:** Alcohol dependence, Primary health care, Family practitioner, Psychiatrist, Continuing medical education

## Abstract

**Background:**

Harmful alcohol consumption in Germany is a serious public health problem: About 7.7 million adults in Germany can be classified as risky alcohol consumers, about 74,000 deaths per year are related to alcohol consumption, and about 1.8 million adults in Germany (18–64 years) are classified as alcohol dependent. A treatment rate of 9% of all alcohol dependent patients in Germany implies a lack of supply and misuse of medical care. The aim of the study was to examine whether family practitioners (FPs) and psychiatrists have sufficient skills to diagnose and treat patients with alcohol problems.

**Methods:**

A total of 6324 FPs and psychiatrists in the states of Saxony and Rhineland-Palatinate in Germany were invited to participate in this survey. Nine hundred seventy-four participants (90.3%/FPs) could be included in the statistical analysis (response rate: 14.3%/FPs, 21.6%/psychiatrists). Data was analysed descriptively and logistical regressions were used to identify predictors for physicians’ ability to feel adequately trained to diagnose and treat patients with alcohol problems.

**Results:**

In comparison to psychiatrists, less FPs reported feeling sufficiently trained to counsel patients with alcohol problems (81.5% vs. 44.8%). Regression analysis revealed that FPs who felt not adequately trained had less experience with patients with alcohol dependence (OR 7.4), had attended fewer hours on alcohol addiction in continuing medical education (OR 4.8), and were more likely to be female (OR 1.9). A minimum of 10 h of training was associated with improved self-assessed competence.

**Conclusion:**

Harmful drinking is a serious public health problem, and patients with alcohol dependence represent a large and demanding patient group in primary health care setting. Our study shows that the lack of training is a severe barrier in the work with this patient group in the primary care setting.

## Background

Alcohol is socially accepted as part of the daily life by many people in European culture. However, consumption of alcohol has become the third highest risk factor for disease and premature mortality in Europe after tobacco consumption and high blood pressure [1, 2]. The annual per capita consumption of pure alcohol among the general population of people > 15 years in 2014 averaged 10.6 l in the European Union vs. 11 l in Germany [3]. The estimated prevalence of alcohol dependence (ICD-10 F10.2: mental and behavioural disorders due to alcohol: dependence syndrome) is 14.6 million adults in Europe [4]. In Germany, 1.8 million (3.4%) of people at the age of 18–64 years are alcohol dependent (4.8% men, 2.0% women) [5]. In addition, approximately 7.8 million German adults consume alcohol in a risky way [6] (> 24 g alcohol/male and > 12 g alcohol/female per day) [7]. It is known that risky alcohol habits often start in adolescence [8].

Annually, 74,000 people die in Germany as a result of increased alcohol consumption [9]. In addition, the harmful use of alcohol (ICD-10, F10.1: mental and behavioural disorders due to alcohol: harmful use), meaning detected consequential psychological or physical harm, can lead to other diseases such as alcoholic liver cirrhosis, pancreas carcinoma and other types of cancer as well as alcohol-related injuries and suicides [10]. The average age at the time of death is about 20 years lower among people with alcohol dependence than among the general population: among women it is 60 years and among men 58 years [11].

Hospital statistics reveal enormous problems caused by alcohol as well: the diagnosis “mental and behavioral disorders due to alcohol” was the second most frequent single diagnosis in German hospitals in 2012 [12]. The numbers for in-patient treatment of alcohol-related illnesses have increased considerably in Germany over the past 18 years. While in 1994 there were 205,733 cases of mental or behavioral alcohol abuse, in 2012 this number had increased to 345,034 cases [13], corresponding to an increase of 68% [14].

Considering economic data, it is estimated that the total costs for the German health system due to alcohol abuse are € 27.6 billion per year. Direct costs are calculated at € 7.4 billion. In addition, costs of € 2.6 billion due to damage to property, crime traffic accidents etc. have to be taken into account. Alcohol-related accidents at work gain an extra € 1 billion, and indirect costs are even higher: mortality losses, lost work productivity, disability, early retirement etc. lead to a total amount of approximately € 16.6 billion [15].

At the same time, primary health care setting often misses the patients with alcohol dependence. In Europe, the treatment quote of about 20% of patients with the psychiatric diagnosis “alcohol dependence” is the lowest among all the psychiatric diagnoses [16, 17]. The estimated treatment rate of only 9% indicates an insufficient and inadequate supply of medical care for alcohol dependent patients in Germany [18]. Thus, among the diseases of the central nervous system, alcohol dependence is the disease with the highest proportion of untreated patients in Germany [16, 19]. Based on an earlier German study, 80% of patients with alcohol dependence seek medical advice in the course of 1 year - in patients with alcohol abuse the proportion is 67% [20]. The critical point is early detection, diagnosis and treatment of patients with alcohol dependency, where especially family practitioners play an initial role.

Education and training are elementary requirements for early detection, diagnosis and treatment of alcohol dependent patients. Various studies indicate that further education and training do not adequately take into account alcohol dependency, and that the ability to handle this sensitive issue and to initiate adequate diagnostics and therapy in time is insufficiently conveyed [21, 22]. A former survey showed that 61.4% of German medical students received no or only a 1–2 h lesson on the topic. Only 13.7% of the students referred to more than 5 h of lessons [23].

The aim of the study was to examine whether family practitioners (FPs) and psychiatrists have sufficient skills to diagnose and treat patients with alcohol problems.

## Methods

The data used in this study was based on a cross-sectional survey on the health care of alcohol dependent patients in primary care in Germany, conducted June to October 2013. The survey aimed to reach all accredited family practitioners and psychiatrists in the federal states of Saxony and Rhineland-Palatinate in Germany (*n* = 6324). These two states were selected based on the structural equity of these regions. All invited physicians were registered at the Association of Statutory Health Insurance Physicians (AHIP) or at the State Chamber of Physicians. The questionnaire and the response envelope were sent by post and a total of 974 questionnaires were returned, resulting in an overall response rate of 15.4% (14.3% in the family practitioners and 21.6% in the psychiatrists group).

### Survey instrument

The questionnaire was developed on the basis of expert interviews. The first structured interview, supported by a group communication manual, was conducted as a group interview with a group of 20 family practitioners and psychiatrists from Saxony. Three further interviews were conducted with individual physicians. During the interviews, the items of the questionnaire were specified and adapted to describe the care reality. The questionnaire included closed-ended questions as well as three to five-point rating scales.

This survey assessed diagnostic and communication strategies that physicians use to identify patients with alcohol dependence. Furthermore, it assessed which barriers and potentials were seen in treating alcohol patients in the physicians’ setting and whether they felt adequately educated, trained, motivated and legitimised to work with these patients. Moreover, the questionnaire included questions on demographic and physician-related characteristics. In the cover letter, anonymity was assured and a helpline was set up for further questions. Personal data such as name or address were not asked or stored; participation was voluntary. The study was approved by the Dresden District Chamber of Physicians (Kreisärztekammer Dresden) stating ethical approval not being necessary for this study, since no patient data was collected. Collection of self-reported data by physicians is in accordance with the German professional regulations for physicians,

### Data analysis

Data were analysed by using SPSS Statistics version 21. Excluded from the analysis were questionnaires without marked answers on the statement “I am adequately trained and educated to work with alcohol dependent patients”. After exclusion of these cases, a total of 748 data sets could be included into the analysis. For a description of the sample, first a descriptive evaluation using contingency tables took place. Chi-square tests were carried out. A *p*-value of < 0.05 was considered significant in all analyses. Conclusively, logistic regressions were performed in which all variables that showed significant results in the bivariate analysis were included “backwards” in the regression equation.

Mann and Whitney χ^2^-tests and U-tests were used to study differences. Finally, logistical regressions were used to identify those variables that influence physicians’ inability to feel adequately trained to work with patients with alcohol dependence or with risky drinking behaviours. A p-value of < 0.05 was considered significant in all analyses. In terms of exploratory data analysis, the p-value was used to show abnormalities in the reality of care.

### Study variables

In the study, the self-assessment of the personal educational and training state represents the dependent variable. To capture the target variables, physicians were asked to indicate whether they feel adequately trained and educated to work with alcohol problems. The answer categories comprised of “applies”, “does not apply” as well as “don’t know”. As independent variables, demographic as well as physician–related features (i.e. occupational years, time required for further education, number of patients, practice location (urban/rural) were selected for the model.

## Results

### Sample description

Most of the responding physicians worked in Saxony (61.6%), the remaining 38.4% were practicing in Rhineland-Palatinate. The gender distribution in the survey was nearly balanced (48.1% male, 51.9% female). The majority of participants reported working as family practitioners (90.3%) and were older than 49 years (66.2%). About half (51.6%) of the physicians were working in rural areas and the other half (48.4%) in urban areas. 71.3% of participants had graduated from medical school more than 20 years ago, 43.5% were practicing medicine for more than 20 years. More than half of the responding physicians reported treating 1000 patients or more quarterly. About one quarter (27.4%) of the physicians had attended fewer than 4 h continuing medical education on alcohol dependency. Only a small percentage (4.3%) had achieved the board certificate “treating addiction in primary care” (Table 1).

**Table 1:** Characteristics of study participants

Variable			*N*	%
*Gender*			*965*	*100*
Male			464	48.1
Female			501	51.9
Missing values			9	
*Age*			967	*100*
< 40 years			93	9.6
40–44 years			93	9.6
45–49 years			141	14.6
50–54 years			224	23.2
55–59 years			178	18.4
60–64 years			118	12.2
> 64 years			120	12.4
Missing values			7	
*Federal state*			*970*	*100*
Saxony			598	61.6
Rhineland-Palatinate			372	38.4
Missing values			4	
*Medical speciality*			*936*	*100*
Family practitioner			845	90.3
Psychiatrist			91	9.7
Missing values			38	
*Practise location*			*965*	*100*
Rural area			498	51.6
Urban area			467	48.4
Missing values			9	
*Physician in private practice since*			*964*	*100*
< 11 years			*307*	*31.8*
11–20 years			*238*	*24.7*
> 20 years			*419*	*43.5*
Missing values			10	
*Patients treated per quarter*			*959*	*100*
Up to 500			77	8.0
Up to 1000			405	42.2
Up to 1500			355	37.0
> 1500			122	12.7
Missing values			15	
*Continuing medical education “alcohol dependency”*			*961*	*100*
< 4 h			*263*	*27.4*
4–10 h			*206*	*21.4*
> 10 h			*266*	*27.7*
Do not know			*226*	*23.5*
Missing values			13	
*Board certificate “treating addiction in primary care”*			*970*	*100*
Yes			42	4.3
No			928	95.7
Missing values			4	

There was a significant gender difference with regard to perceived education and training concerning the treatment of alcohol dependent patients: 57.1% of male vs. 41.9% of female physicians reported they “feel adequately trained” to work with alcohol dependent patients (χ^2^ test, *p* < 0.001, Table 2). The age group of 55 years and older felt better trained compared to younger participants (χ^2^ test, *p* < 0.021, Table 2).

**Table 2:** Bivariate relationships between demographic factors, physician-related features and perceived training competence

Variable	*n*	‘I am adequately trained and educated to work with alcohol dependent patients.’		*p*-value
		Does apply	Does not apply	
*Gender*				
Male	354	202 (57.1%)	152 (42.9%)	< 0.001
Female	389	163 (41.9%)	226 (58.1%)	
*Age*				
< 40 years	60	29 (48.3%)	31 (51.7%)	0.021
40–44 years	69	30 (43.5%)	39 (56.5%)	
45–49 years	107	53 (49.5%)	54 (50.5%)	
50–54 years	173	67 (38.7%)	106 (61.3%)	
55–59 years	139	76 (54.7%)	63 (45.3%)	
60–64 years	96	54 (56.3%)	42 (43.8%)	
> 64 years	99	57 (57.6%)	42 (43.8%)	
*Federal state*				
Saxony	450	221 (49.1%)	229 (50.9%)	n.s.
Rhineland - Palatinate	295	146 (49.5%)	149 (50.5%)	
*Medical speciality*				
Family practitioner	634	284 (44.8%)	350 (55.2%)	< 0.001
Psychiatrist	81	66 (81.5%)	15 (18.5%)	
*Practise location*				
Rural area	374	170 (45.5%)	204 (54.5%)	n.s.
Urban area	368	193 (52.4%)	175 (47.6%)	
*Years after graduation from medical school*				
<11 years	40	16 (38.1%)	26 (61.9%)	n.s.
11–20 years	157	82 (50.9%)	79 (49.1%)	
>20 years	536	269 (49.6%)	273 (50.4%)	
*Number of patients diagnosed as ICD-10: F10.1 in 2012*				
0–6	143	46 (32.2%)	97 (67.8%)	< 0.001
7–12	179	71 (39.7%)	108 (60.3%)	
13–24	145	76 (52.4%)	69 (47.6%)	
25–49	153	86 (56.2%)	67 (43.8%)	
> 49	102	78 (76.5%)	24 (23.5%)	
*Number of patients diagnosed as ICD-10: F10.2 in own practise*				
0–6	215	77 (35.8%)	138 (64.2%)	< 0.001
7–12	213	91 (42.7%)	122 (57.3%)	
13–24	144	81 (56.3%)	63 (43.8%)	
25–49	91	52 (57.1%)	39 (42.9%)	
> 49	66	56 (84.8%)	10 (15.2%)	
*Continuing medical education on “alcohol dependency”*				
0–3 h	196	54 (27.6%)	142 (72.4%)	< 0.001
4–10 h	141	47 (33.3%)	94 (66.7%)	
> 10 h	233	170 (73.0%)	63 (27.0%)	
*Board certificate “treating addiction in primary care”*				
Yes	37	33 (89.2%)	4 (10.8%)	< 0.001
No	709	334 (47.1%)	375 (52.9%)	

One major difference was seen between the physician groups (χ^2^ test, *p* < 0.001, Table 2). The majority of psychiatrists (81.5%) felt adequately trained to work with alcohol dependent patients, while only 44.8% of family practitioners agreed on this.

Physicians treating several patients with alcohol dependence syndrome (ICD-10 F10.2) felt sufficiently trained for working in this field (χ^2^ test, *p* < 0.001, Table 2): Only 35.8% of the physicians with up to six cases per year consider themselves to be adequately trained, while 84.8% of the physicians with more than 50 cases per year felt adequately qualified. A similar picture emerges by looking at the number of treated cases of harmful use of alcohol (ICD-10 F10.1). In addition, physicians having invested more time in continuing medical education on alcohol dependence, or holding the additional board certificate “treating addiction in primary care”, felt significantly better trained than others (χ^2^ test, *p* < 0.001, Table 2).

No significant differences could be found in physicians’ assessments between the included federal states: Almost half of both the Saxon and the Rhineland-Palatinate physicians felt well trained to work with alcohol dependent patients. Moreover, there was no significant relationship between work experience in terms of years after graduation or time after settlement or the location of the practice (urban vs. rural) and the assessment of alcohol-specific education and training.

### Multivariate analysis

In the logistic regression model, the following factors were identified determining whether a physician does not feel adequately trained and educated to work with alcohol dependency (Table 3): The most influential determinant is the number of treated cases per year of alcohol dependence syndrome (ICD-10 F10.2). Among physicians treating an average of up to six cases a year, the likelihood of not feeling sufficiently trained increases around 5.2 times compared to physicians who treat more than 50 cases per year. Another important factor is continuing medical education: the fewer the hours a physician spent on training on “alcohol dependence” after graduation, the higher is the risk that he does not feel adequately trained to work with this kind of patients: 27.4% of the physicians answered they had had “none” or “less than 4 hours”, and 21.4% between 4 to 10 h of continuing education in this field. Regression analysis selected < 4 or 4 to 10 h of training time as an important predictor for not feeling sufficiently trained and educated (OR 5.15 vs. 4.88) compared to those who spent 11 or more hours on alcohol dependence training.

**Table 3:** Logistic regression: Factors that influence the likelihood that physicians do not feel adequately trained and educated to care for patients with alcohol dependence

Predictors	*p*-value	Odds Ratio
*Number of patients diagnosed as ICD-10: F10.2 “Mental and behavioural disorders due to alcohol: dependence syndrome”*		
0–6	0.001	5.17
7–12	0.001	3.74
13–24	0.016	2.80
25–49	0.051	2.43
> 49	RC	–
*Gender*		
Male	RC	–
Female	0.001	2.00
*Medical speciality*		
Psychiatrist	RC	–
Family practitioner	0.001	3.05
*Continuing medical education on “alcohol dependency”*		
0–3 h	0.001	5.15
4–10 h	0.001	4.88
> 10	RC	–
Do not know	0.003	2.05
*Board certificate “treating addiction in primary care”*		
Yes	RC	–
No	0.031	3.58

*RC* Reference Category

Physicians without the additional board certificate “treating addiction in primary care” had a 3.6 times higher risk of not feeling adequately trained. Compared to psychiatrists, family practitioners had a higher likelihood of not feeling adequately trained and educated to work with alcohol dependent patients (OR 3.1).

The same holds for female physicians if compared with males. Female physicians had a 2-fold risk in comparison to their male colleagues of not feeling well prepared to work with alcohol dependent patients. The Nagelkerke-R^2^ value of 0.347 indicated a medium-sized explanatory power of the logistic regression model.

## Discussion

Our survey on family practitioners and psychiatrists in two German federal states indicates that a large proportion of physicians, especially family practitioners, do not feel sufficiently trained and educated to ensure an effective and efficient care for patients with alcohol dependence. In comparison, general practitioners in the United Kingdom seem to feel much better educated and trained to treat patients with alcohol problems than their German counterparts. The positive assessment among British FPs regarding being adequately educated trained was twice as high as in our sample [24].

Our study shows that attending continuing medical education of alcohol dependency and achieving the board certificate ‘treating addiction in primary care’ is associated with feeling more adequately trained to diagnose and treat patients with alcohol problems, as also stated by van Boekel et al. [25]. Also the amount of training hours was shown relevant: According to our data, a minimum of 10 h of training was associated with improved self-assessed competence. However, about one quarter of the physicians reported that they have had “no” or “less than 4 hours” and one fifth “4 to 10 hours” training time on the topic of alcohol dependence after completing their medical studies. This indicates there is a great potential for optimizing the supply of training to address the specific needs of care regarding the patients with alcohol dependence in the primary care setting.

With regard to gender differences among physicians treating patients with alcohol problems, female physicians in our study were more critical than males regarding their self-assessment of their competences and estimated being less sufficiently trained and educated to provide an effective and efficient treatment for patients. The more critical self-reflection among female physicians is also known from elsewhere [26, 27]. Nevertheless, advanced training should be offered to all family practitioners, especially in rural areas.

Training of physicians is elementary for improving the outcomes regarding early detection, diagnosis and treatment of patients with alcohol problems. There is a number of further barriers to overcome, such as patient motivation or inadequate payment, that are not that modifiable as organisation of training. The supply of an adequate amount of training is necessary, but the contents of training should be more in focus [28]. Training will be more acceptable and effective if it is tailored to the needs of physicians in the primary care setting. Studies suggest that training should include physician-patient communication as an essential factor for early recognition of alcohol dependency. Beside more focus on motivational interviews in continuing medical education, physicians should be trained to employ brief screening instruments [29, 30].

## Conclusion

Harmful drinking is a serious public health problem and the number of undiagnosed cases is high. Patients with alcohol dependence represent a large and demanding patient group in primary health care setting. Our study shows that the lack of training is a severe barrier in detecting, diagnosing and treating patients with alcohol problems in the primary care setting. Based on the study results, the quality and quantity of the education and training of physicians regarding alcohol dependency should be increased to improve physicians’ ability to competent and result-oriented care for a large group of patients with alcohol problems in the primary care setting. Training of a minimum of 10 h is recommended in order to improve diagnostic and therapeutic competences regarding patients with alcohol problems.

### Limitations

Although the response rate of our survey (15%) is lower compared to other surveys with smaller collectives of physicians, the large sample size of the population of invited physicians indicates sufficient representativeness of data [24, 31]. It can be assumed that the practitioners who participated in the survey do have at least some interest in this topic. Accordingly, it is possible that the quality of care might be more negative than suggested by this analysis. As in almost every survey without full census, the selection bias cannot be ruled out.

## Data Availability

The questionnaire (in German) can be requested from the authors. The datasets supporting the conclusions of this article are stored in electronic and paper form at the Department of Health Sciences / Public Health at the Technische Universität Dresden in accordance with the applicable data protection regulations. The datasets generated and analysed during the current study are not publicly available due to the data protection regulations but are available from the corresponding author on reasonable request..

## Abbreviations

AHIP: Association of Statutory Health Insurance Physicians
F10.1: Mental and behavioural disorders due to alcohol: harmful use of alcohol
F10.2: Mental and behavioural disorders due to alcohol: alcohol dependence syndrome
FPs: Family practitioners
ICD-10: International Classification of Diseases and Related Health Problems, 10th revision
OR: Odds ratio

## References

[1] Anderson P (2006). Baumberg. Alcohol in Europe. A Public Health Perspective.

[2] Linardakis M, Papadaki A, Smpokos E, Komninos Y, Philalitis A (2014). Multiple behavioral risk factors for chronic diseases in adults aged 50+: regional differences across eleven European countries. J Public Health.

[3] Schaller K, Kahnert S, Mons U (2017). Alkoholatlas Deutschland 2017.

[4] Wittchen HU, Jacobi F, Rehm J (2011). The size and burden of mental disorders and other disorders of the brain in Europe 2010. Eur Neuropsychopharmacol.

[5] Pabst A, Kraus L, Gomes de Matos E, Piontek D (2013). Substanzkonsum und substanzbezogene Störungen in Deutschland im Jahr 2012. Sucht.

[6] Rummel C, Lehner B, Kepp J, editors. Daten, Zahlen und Fakten. In: Deutsche Hauptstelle für Suchtfragen e.V. Jahrbuch Sucht 2017. Lengerich: Pabst Science Publishers; 2017.

[7] Seitz HK, Bühringer G, Mann K (2008). Grenzwerte für den Konsum alkoholischer Getränke: Empfehlungen des wissenschaftlichen Kuratoriums der DHS. Deutsche Hauptstelle für Suchtfragen. Jahrbuch Sucht 2008.

[8] Soellner K, Göbel K, Scheithauer H, Bräker A-B (2014). Alcohol use of adolescents from 25 European countries. J Public Health.

[9] Gaertner B (2013). Alkohol – Zahlen und Fakten zum Konsum. Jahrbuch Sucht.

[10] Babor T, Caetano R, Casswell S, Edwards G, Giesbrecht N, Graham K, Grube J, Gruenewald P, Hill L, Holder H, Homel R, Österberg E, Rehm J, Room R, Rossow I (2005). Alkohol – Kein gewöhnliches Konsumgut.

[11] John U, Rumpf HJ, Bischof G, Hapke U, Hanke M, Meyer C (2013). Excess mortality of alcohol-dependent individuals after 14 years and mortality predictors based on treatment participation and severity of alcohol dependence. Alcohol Clin Exp Res.

[12] Deutsche Hauptstelle für Suchtfragen (DHS) (Hrsg) (2015). Daten/Fakten, Alkohol.

[13] Statistisches Bundesamt. Diagnosedaten der Patienten und Patientinnen in Krankenhäusern 2012. Wiesbaden: Statistisches Bundesamt; 2013. Fachserie 12, Reihe 6.2.1.

[14] Gaertner B, Meyer C, John U, Freyer-Adam J (2014). Alkohol – Zahlen und Fakten zum Konsum. Deutsche Hauptstelle für Suchtfragen e.V. (Hrsg) Jahrbuch Sucht 2014.

[15] Bartsch G, Merfert-Diete C, Badura B, Ducki A, Schröder H, Klose J, Meyer M (2013). Alkoholabhängigkeit und riskanter Alkoholkonsum. Berlin Fehlzeiten-Report.

[16] Kohn R, Saxena S, Levav I, Saraceno B (2004). The treatment gap in mental health care. Bull World Health Organ.

[17] Rehm J, Allamani A, Elekes Z, Jakubczyk A, Manthey J, Probst C, Struzzo P, Della Vedova R, Gual A, Wojnar M (2015). Alcohol dependence and treatment utilization in Europe – a representative cross-sectional study in primary care. BMC Fam Pract.

[18] Rehm J, Shield KD, Rehm MX, Gmel G, Frick U (2012). Alcohol consumption, alcohol dependence and attributable burden of disease in Europe: potential gains from effective interventions for alcohol dependence.

[19] Alonso J, Angermeyer MC, Bernert S (2004). Use of mental health services in Europe: results from the European study of the epidemiology of mental disorders (ESEMeD) project. Acta Psychiatr Scand Suppl.

[20] Rumpf HJ, Meyer C (2000). Inanspruchnahme suchtspezifischer Hilfen von Alkoholabhängigen und -missbrauchern: Ergebnisse der TACOS Bevölkerungsstudie. Sucht.

[21] Strobel LKI. Eine Studie zur Ausbildungssituation deutscher Medizinstudenten hinsichtlich Tabak- und Alkoholabhängigkeit: Universität Göttingen; 2012. Dissertation. http://d-nb.info/1044047542/34. Accessed 13 Feb 2015.

[22] Voigt K, Twork S, Mittag D, Göbel A, Voigt R, Klewer J, Kugler J, Bornstein SR, Bergmann A (2009). Consumption of alcohol, cigarettes and illegal substances among physicians and medical students in Brandenburg and Saxony (Germany). BMC Health Serv Res.

[23] Hoffmann T, Voigt K, Schlisske L, Riemenschneider H, Bergmann A, Kugler J (2015). How fit are medical students from Dresden to treat alcohol-related disorders?. Z Allg Med.

[24] Wilson GB, Lock CA, Heather N, Cassidy P, Christie MM, Kaner EFS (2011). Intervention against excessive alcohol consumption in primary health care: A survey of GPs’. Attitudes and practices in England 10 years on. Alcohol Alcohol.

[25] van Boekel LC, Brouwers EP, van Weeghel J, Garretsen HF (2014). Healthcare professionals’ regard towards working with patients with substance use disorders: comparison of primary care, general psychiatry and specialist addiction services. Drug Alcohol Depend.

[26] Cooper KM, Krieg A, Brownell SE (2018). Who perceives they are smarter? Exploring the influence of student characteristics on student academic self-concept in physiology. Adv Physiol Educ.

[27] Voigt K, Schübel J, Spornraft-Ragaller P, Bergmann A, Riemenschneider H (2017). Sexuell übertragbare Infektionen – Thema für die Hausarztpraxis? [sexually transmitted infections – an issue for family practitioners?]. Z Allg Med.

[28] Fitzgerald N, Commentary on Anderson (2016). The question is not just whether to incentivize and train practitioners on alcohol screening and brief advice, but how?. Addiction.

[29] Mueller G, Schumacher P, Wetzlmaier J, Pallauf M (2016). Screening questionnaires to identify problem drinking in the primary care setting: a systematic review. J Public Health.

[30] Anderson P, Kaner E, Keurhorst M, Bendtsen P, Steenkiste BV, Reynolds J (2017). Attitudes and learning through practice are key to delivering brief interventions for heavy drinking in primary health care: Analyses from the odhin five country cluster randomized factorial trial. Int J Environ Res Public Health.

[31] Unrath M, Zeeb H, Letzel S, Claus M, Pinzón LCE (2012). The mental health of primary care physicians in Rhineland-Palatinate, Germany: the prevalence of problems and identification of possible risk factors. Dtsch Arztebl Int.

